# Application of six sigma and the system thinking approach in COVID-19 operation management: a case study of the victorian aged care response centre (VACRC) in Australia

**DOI:** 10.1007/s12063-022-00323-2

**Published:** 2022-10-07

**Authors:** Sandeep Jadhav, Ahmed Imran, Marjia Haque

**Affiliations:** 1 Information, Technology (IT) & Systems, University of Canberra, Canberra, Australia; 2 Department of Defence, Government of Australia, Melbourne, Australia

**Keywords:** COVID-19 pandemic, Aged care, Six Sigma, Systems thinking approach, Organizational excellence

## Abstract

COVID-19 has posed many unique and critical challenges in various contexts and circumstances. This often led the stakeholders and decision-makers to depart from traditional thinking and the business-as-usual processes and to come up with innovative approaches to tackle various mission-critical situations within a short time frame. In this paper, a real-life case study of COVID-19 operation management following a multi-disciplinary, multi-stakeholder novel integrated approach in aged care facilities in Victoria, Australia, is presented which yielded significant and positive outcomes. The purpose of the intervention was to develop an integrated system performance approach through the application of various quality management tools and techniques to achieve organizational excellence at the aged care centers. The case involved the use of mathematical models along with statistical tools and techniques to address the specific problem scenario. A system-wide management plan was proposed, involving various agencies across several residential aged care facilities during the pandemic. A three-step methodological framework was developed, where Six Sigma, a system thinking approach, and a holistic metric were proposed to manage the value chain of the pandemic management system. The experimental result analyses showed significant improvement in the management process, suggesting the validity and potential of this holistic approach to stabilize the situation and subsequently set the conditions for operations excellence within the sectors. The model offers new insight into the existing body of knowledge and offers an efficient approach to achieving operational excellence in any organization or business regardless of its type, shape and complexity, which can help practitioners in managing complex, mission-critical situations like a pandemic.

## Introduction

The novel Corona Virus Disease 2019 or COVID-19 has posed a new and unexpected global challenge in this twenty-first century. In many cases, this required innovative and emergency responses to bring the escalating situation under control in different circumstances and contexts. Most sectors in all countries around the globe have been affected by the pandemic. The COVID-19 pandemic has brought about many unprecedented challenges and threats to the world's humanity and the lives and well-being, particularly of older people (Chee 2020). For example, Australia stood out as an exemplar for its response during the first few months of the pandemic, however later, approximately 75% of the country’s COVID-19 deaths occurred in residential aged care facilities, especially in the state of Victoria (Cousins 2020). Therefore, researchers and practitioners were forced to think differently to deal with the crisis of this sector while being motivated by the saying of Napolina Hill, “In every adversity lies the seed of an equal or greater opportunity”. Doctors, nurses, hospital management systems, and governments of various countries kept no stone unturned to contain the rapid spread of the virus and to save lives. COVID-19 management can be viewed as a classic example of a complex problem that requires collective effort and input from multiple stakeholders within a broader organizational perspective. However, there was a notable absence of efficient management coordination and a lack of appropriate planning across various involved parties which caused much chaos during pandemic management crises in various countries (Imran 2020). Thus, a study examining the development of innovative approaches such as an integrated management plan involving related stakeholders or agencies became necessary.

While it is difficult to develop and implement a standard approach for dealing with pandemics like COVID-19, current research demonstrates considerable gaps in this sphere and emphasized the importance of establishing an efficient management plan to contain a crisis like a pandemic (Jadhav et al. 2021; Reed 2021). Few studies have examined the management of the COVID-19 crisis as a system-wide performance achievement process (Hundal et al. 2021a), (Kuiper et al. 2021), (Hassan et al. 2020; Zięba 2021). Also, the COVID-19 situation being highly contextual and volatile, a standard framework or “one size fits all” approach for its management and containment appeared to be difficult to develop. The fluid and changing circumstances with a lot of uncertainties also did not allow researchers to address this issue more objectively with detailed and well-planned research. Hence the best practices, real-life cases and experiments became a major source of research and experiment to derive new knowledge in this area.

In practice, most organizations have attempted a significant number of improvement initiatives such as total quality, reengineering, restructuring, and teams, with very mixed success. In many cases, these initiatives have been adopted without being part of an improvement strategy, but as part of a series of ‘Adhoc’ decisions. Against this backdrop, this study, motivated by real-life experiences in implementing an organization-wide management plan for COVID -19 in aged care facilities in Victoria, Australia was chosen to experiment and test the proposition. In this context, achieving operational excellence (OE) became the key to success which is required to be implemented across healthcare sectors and/or hospitals, in order to manage the ongoing crisis. OE methods can be used to protect patient and public health to ensure safety and conquer challenges (McDermott et al. 2021). On the other hand, researchers have attempted to implement six-sigma approaches, in handling the COVID-19 crisis across various countries (Salentijn et al. 2021), (Raja Mohamed et al. 2021). Lean Six Sigma (LSS) has also been applied to mitigate the disruption that occurred in healthcare industries due to the COVID-19 disaster (Hundal et al. 2021b), (Muhammad et al. 2022). However, studies integrating various units responsible for pandemic management, thus ensuring a holistic planning and implementation approach to handle the crisis, are scarce.

This study explored the deeper ‘pre-conditions’ required during emergencies for organizational-make especially when uncertainty and ambiguity are rampant. It then examined the management system and measurement metric to assess organizational wellness and understand variability to obtain sustained performance improvements. A holistic management system, tools, and techniques have been proposed that will not only be applied during emergencies e.g., Tsunami, pandemics, etc. but also applicable to steady-state within wider businesses. This approach to management is different from the ‘Adhoc’ approach, and executives or business leaders can assess and predict risks, which is one of the major novelties of this study.

Aged care is considered one of the important sectors in many countries. Ibrahim (2020) highlighted the importance of understanding the existing gaps in the aged care sector of Australia and the necessity to bridge these for pandemic management. This paper filled this void across different aged care facilities in Victoria during the COVID-19 containment process by offering unique management processes and performing statistical analyses. In this case of aged care facility management in Victoria, the vast range of potential combinations of variable factors became virtually impossible to manually solve. Statistical tools and analyses in such circumstances have proven to be useful. The Design of Experiments (DoE) (Cox and Reid 2000) is a statistical tool that provides an approach for optimizing the inputs of greatest influence, understanding the system-effect of their variability, and addressing any uncertainty, especially in a resource- and time-constrained environment. Accordingly, in the case of COVID-19 control and management operations in residential aged care facilities (RACFs), various quality management techniques and statistical analyses were considered as operation management tools and the efficacy of these tools for achieving OE and capturing the complexity of the issue was tested. As such, the Six Sigma tool and systems thinking approach with a holistic metric were considered as the catalysts to efficient management of the COVID-19 crisis.

Accordingly, guided by Yin’s (2017) case study approach, most suitable to investigate a complex phenomenon, the paper addresses the following research questions:

### R1:

How can the COVID-19 outbreak be predicted and prevented, as opposed to detect and prevent approach and how the assumptions can be measured and validated?

### R2:

How the conditions within Victorian Aged Care Centres can be improved and how the control mechanisms can be established through One Metric That Matters the Most (OMTM)?

To answer these research questions, the paper presents a real-life case study involving 21 agencies and authorities who were directly or indirectly involved in the pandemic management process in aged care facilities in Victoria. The study explored how the leaders across the various involved agencies responded to the COVID-19 crisis in aged care centers and explains how the management frameworks, predictive approaches to solution design, and Six Sigma principles were applied in combination to achieve the desired outcome. Drawing insight from the case study, the objective of this paper was to develop an integrated management system and associated metrics using various 21st-century quality management tools as well as a set of guiding principles that are universal to the achievement of OE. In the study, a performance gap was identified to establish a management framework and OMTM during uncertainty and ambiguous situations of the COVID-19 outbreak in Victorian aged care centres. The final outcome of the study is to propose a strategic framework to organizations and bodies like the Commonwealth of Australia (Department of Health) within the aged care sectors in order to create a culture of organizational excellence using the Six Sigma methodology and system thinking approaches. The framework includes a management system and most importantly develops a single metric to assess enterprise risk or wellness. This will preposition the aged Care Sectors to take timely responses or countermeasures during the future pandemic and also manage the current enterprise-wide risks. Whilst the study is focused on the aged care sector, the theory can be applied to any organization or business regardless of its type, shape and complexity.

The remainder of the paper is organized as follows. Section 2 presents a detailed literature review on the research topic. A description of the particular case under study is presented in Sect. 3 and the detailed research methodology is outlined in Sect. 4. Section 5 highlights the experimental findings. Finally, the results are discussed, conclusions are drawn, with the limitations of the study are considered in Sect. 6.

## Literature review

This section presents an up-to-date literature review with a focus on understanding the current knowledge in the field and identifying potential gaps. The review starts by describing several major quality management tools, for example, Lean, Six Sigma, Lean Six Sigma and the system thinking approach. Then, the review focuses on the application of these tools in practical cases, especially in the healthcare industry. Finally, the application of these tools for the COVID-19 management crisis is examined.

As part of process improvement, continuous improvement (CI) is the process of making ongoing improvements to products, services, programs, or processes that play vital roles in organizations (Lam et al. 2015). Among the various CI methodologies found in the literature, Lean and Six Sigma are two powerful CIs that are widely used and capable of evolving organizational needs through the CI process (Sreedharan and Sunder 2018).

The Lean concept originated within the Japanese automobile industry following the Second World War and was primarily focused on the elimination of Muda, or waste (Ohno 1988). Waste can be defined as any non-value-added activity that does not create value for the end customer (Cudney et al. 2013). The focus is on non-value-added waste elimination and the seven wastes of transport, inventory, motion, waiting, overproduction, over-processing, and defects, which are all vital in various organizations, including healthcare. The non-value activities may comprise up to 95% of activities in healthcare operations, meaning there is scope for substantial improvement (Gowen et al. 2012). The basic principles of the Lean philosophy are the minimization of waste, increasing the speed of all processes across the enterprise, and improving the organization’s performance. A Lean system consumes fewer resources, brings better results, and provides increased benefits for the business to achieve competitive advantages (Hines et al. 2004; Wickramasinghe and Wickramasinghe 2011).

In contrast, Six Sigma is a data-driven statistical tool used to reduce errors or defects due to excess variation within processes (Antony 2012). Specifically, it is used to reduce and control variation in a process so that the process can be improved to meet its target. By definition, sigma or standard deviation represents the variability in a parameter characteristic (e.g., a process parameter, delivery time, response time, etc.) requiring control. According to the properties of a normal distribution, a Six Sigma level process has controlled variation within one-half of the allowed variation limits. When undesirable variation is removed and natural variation is predictable, the outcome can be planned with certainty, which is implied by Six Sigma. Usually, the Six Sigma methodology employs a ‘Define, Measure, Analyse, Improve, And Control’ (DMAIC) or a ‘Plan, Do, Check, Adjust’ (PDCA) approach to deal with problems with unknown solutions, particularly when the root causes need to be discovered (Antony et al. 2018). Six Sigma is a useful tool for quality management processes to achieve business process improvements. It has been implemented successfully by many large manufacturing companies, such as Motorola, GE, and Honeywell. The study of Ismyrlis and Moschidis (2018) demonstrated that companies implementing Six Sigma outperform companies that do not incorporate it.

LSS is the synergistic use of Lean and Six Sigma. As Lean cannot provide statistical control for a process alone, and Six Sigma cannot improve process speed, an integrated approach combining both can achieve improved results (George 2002). The combined effect of Lean and Six Sigma has been highlighted by many researchers over the past few decades (Antony et al. 2018). The integration of Lean and Six Sigma can achieve better results than those achieved with each individual system alone. The combined LSS strategy integrates both human aspects (such as leadership, customer focus, and cultural change) and process aspects (such as process capability, process management, and statistical thinking) as a process of CI (Antony 2011). LSS is an approach to achieving excellence by adopting systems thinking approach, including inputs, processes, and outputs. Thus, the LSS method using the DMAIC strategy ensures a robust framework to achieve business excellence (Su et al. 2006). Although it is beneficial, the adoption of LSS is not common, and many organizations often face challenges in implementing it (Yadav et al. 2021). Moosa and Sajid (2010) critically reviewed Six Sigma from both an academic and application point of view. They explored and analyzed several critical factors in the implementation of Six Sigma in organizations based on real-life practice and an analysis of the available literature. The study concluded that the success or failure of such programs mostly depends on the implementation approach rather than its contents.

Though LSS was initially used only in the manufacturing industry, it has gained popularity as a managerial tool, especially in the healthcare sector, with several applications over the past few decades. For example, Six Sigma and LSS have been widely used in the healthcare sector as management strategies to improve patient quality and safety (Trakulsunti et al. 2020). Langabeer et al. (2009) examined whether the use of Lean and Six Sigma quality improvement initiatives would actually help organizations in the healthcare industry to achieve their goals. This research provided descriptive results based on a cross-sectional analysis of a sample of hospitals. Similarly, the study of Lifvergren et al. (2010) showed that Six Sigma can be a useful tool to improve healthcare processes. This research was based on an examination of a three-year quality program using Six Sigma in a Swedish hospital group. The authors argued that implementing Six Sigma can confer a 75% higher success rate compared to the effects of other healthcare improvement approaches. In another study, Gowen et al. (2012) examined how process improvement (PI) initiatives mediate the effect of medical error sources on hospital outcomes. The authors explored three PI initiatives: Continuous Quality Improvement (CQI), Six Sigma Initiatives (SSI), and Lean Management Initiatives (LMI).

Lean principles are widely applied in healthcare operations to manage demand and capacity, improve quality, improve safety, improve supplier relations, and reduce costs, thereby improving processes for patient care (Womack and Jones 2017). Several researchers have used LSS tools in hospital management studies. For example, Bhat et al. (2019) studied various LSS tools and techniques in the context of Indian hospitals and showed that the LSS strategy can be effectively applied even in rural hospitals with minimum resource utilization, achieving significant improvements. Scala et al. (2021) and Improta et al. (2020) both implemented an SS methodology based on the DMAIC cycle to reduce the length of hospital stays for patients in a hospital in Italy. As the target of Lean thinking is to reduce waste, whereas Six Sigma aims to reduce variation through statistical analysis and process control, integration of Lean principles with Six Sigma can serve to improve patient satisfaction and outcomes in the healthcare industry (Bhat et al. 2019). Trakulsunti et al. (2020) proposed a roadmap involving the use of the LSS strategy across an organization to reduce medication errors. This roadmap helped healthcare practitioners and professionals to apply LSS in a disciplined, organized, and systematic way to reduce medication errors. The first phase of the roadmap assessed the cultural readiness of the organization to employ LSS. The next phase highlighted the key factors for preparing the organization to implement LSS. The factors included top management commitment, LSS project selection, team formation, and training in the implementation of LSS methodology. In summary, Six Sigma, Lean, and LSS have been used by researchers to achieve OE in the healthcare industry.

Total quality management (TQM) is a management strategy involving top management and other workers within the organization; it is used to achieve a quality focus at all levels of the organization. Systems thinking is considered an important dimension in the implementation of the TQM framework in an organization (Oschman 2017), (Talapatra and Uddin 2019). It is a concept that uses scientific discoveries and instruments to enable a clear understanding of the integrity of phenomena and the achievement of the desired changes (Skaržauskienė and Carlucci 2010), (Talapatra et al. 2019). From a traditional or classical viewpoint, a system can be defined as a combination of two or more elements, in which every element influences the behavior of other elements, and the behavior of each element influences the behavior of the whole (Bertalanffy 1969; Forrester 1975). Thus, this view separates the individual pieces of a system. In contrast, the systems thinking viewpoint emphasizes that a set of elements interact to produce behavior in the whole system of which they are a part of (Skaržauskienė and Carlucci 2010), (Talapatra et al. 2018). Therefore, the systems thinking approach marks a considerable change in the way an organization is traditionally viewed; it involves a change in the organization’s usual perception in which the combination of different components of an organization is considered as the general system (Ershadi and Eskandari Dehdazzi 2019). As such, this approach links various parts of an organization to a single whole in order to organize different activities of an organization into one.

An organization can be viewed as a group of people who work together in a structured way for a shared purpose (Gulick 1937). However, working together in an organized way requires a systems approach to achieve the organizational purpose. Existing research demonstrates that many organizations are realizing the value of implementing process improvement standards, frameworks, and enterprise strategies to achieve business excellence under uncertain and changing environments (Porter and Tanner 2004; Saleh and Watson 2017). There is strong evidence that superior business performance can be achieved through the alignment and integration of different business functions (Chan and Reich 2007; Rahman et al. 2020). Thus, practitioners and researchers are faced with the challenge of understanding how to effectively implement business strategies in order to achieve value and a competitive edge in the market. The commercial business environment is increasingly driven by stakeholder value, customer loyalty, staff retention, corporate governance, market share, and profit against a push to reduce overall costs (Samson 2008). To this end, mathematical model-based experiments are increasingly being used in many organizations to mitigate program risk by identifying early problems in system design or sustainment (Estefan 2007). As knowledge inputs to these systems are critical, many organizations seek specialist subject matter expert (SME) advice to address challenges and develop solutions. Effective systems methodology includes the following four foundations of systems thinking (Gharajedaghi 2019):

- Holistic thinking: where the focus is on the system as a whole; this requires understanding the structure, function, process, and context of the system.
- Operational thinking (dynamic thinking): refers to the system's dynamics, which may involve feedback systems, identification of the effect and growth, measuring stock and flow, etc. These principles create additional value for managing an organization, whereas business systems are seen as interdependent.
- Interactive design: the art of finding differences among things that seem similar and the science of finding similarities among things that seem different.
- Self-organization: this involves movement toward predefined order*.*

Therefore, the significance of systems thinking can be interpreted as an understanding of interrelations that are not associated with linear cause-effect and the identification of processes of change that are not in static states (Senge 1990). For this, a problem should be solved starting from the whole, as one component cannot be affected separately by other components. Systems thinking may help to detect the order in a complex system and to ensure a better understanding of reality. Hence, systems thinking is viewed as a discipline of the ‘structure’ with complex situations (Senge et al. 2007). A famous commentator in the field of systems thinking states: “Systems approaches aim to simplify the process of our thinking about and managing complex realities that have been variously described by systems thinkers as messes, the swamp, wicked problems. Systems thinking provides ways of selectively handling the detail that may complicate our thinking in a transparent manner, in order to reveal the underlying features of a situation from a set of explicit perspectives” (Reynolds and Holwell 2010, p.5). An ideal structure would be to employ a ‘systems thinking approach for management within all sectors with a set of guiding principles that are common to all organizations regardless of their type, shape, size, and complexity. This approach notes that most organizations are typically comprised of closely connected elements, including leadership, strategy, customer engagement, performance management, employee relationship, core business processes, and data management (Jadhav 2019). An integrated systems approach to OE is a broader program of improving and sustaining business performance, in which quality management is embedded (Basu 2009).

However, as mentioned earlier, few studies have examined operation management techniques used to contain COVID-19 crises. It has been challenging to manage healthcare providers with an appropriate operational plan within the rapidly changing, volatile COVID-19 pandemic context. Coordinating various activities across distinct agencies within an extremely short period of time under limited resources has been the most challenging task for authorities. In the published literature, few studies have examined the use of quality management tools in pandemic management. McDermott et al. (2021) studied how OE can play a role in protecting the public against COVID-19. A few studies have also used Six Sigma and LSS as management tools during the pandemic. For example, Bañez et al. (2020) analyzed and identified the factors contributing to the mitigation of COVID-19 transmission in the Philippines using the DMAIC framework. Kuiper et al. (2021) used Six Sigma to study the situation in the Netherlands during the COVID-19 crisis with respect to process improvement efforts. Hundal et al. (2021a, b) investigated how LSS may help mitigate the impact of COVID-19 within healthcare environments. These authors performed semi-structured interviews and the results revealed that personal safety was the primary concern, followed by process redesign and telemedicine. Bhandar et al. (2021) explored how the use of LSS could help the healthcare sector be better prepared during the global pandemic. Their research utilized the LSS tool and the DMAIC approach to develop strategies for community-based hospitals in the Midwestern US under COVID-19 pandemic planning.

In addition, Gonella et al. (2020) examined several methodological approaches that can be used for communication purposes in epidemics. The authors used systems thinking approach to develop a stock-flow diagram for the COVID-19 pandemic. Jackson (2020) suggested how things might have been different had the ‘critical systems thinking’ view of complexity been employed, and a ‘critical systems practice’ approach adopted when preparing for a possible pandemic and responding to it; this study used the Covid-19 pandemic in the UK as an example. In a recent study, Haley et al. (2021) argued that to tackle difficult problems like the COVID-19 pandemic, a systemic view with systems thinking ideas should be explored from different systemic perspectives.

However, very few studies have developed a system-wide integrated management plan using quality management tools to tackle the COVID-19 crisis. In addition, there has been little focus on aged care facility planning in Australia during the COVID-19 pandemic. Viray et al. (2021) studied six RACFs in Victoria and observed residential in-reach (RiR) services within these facilities. RiR services in Victoria typically consist of small teams of senior medical doctors and nurse specialists operating out of each public hospital network. The researchers collected data on the cumulative proportion of residents who tested positive for COVID-19 over 21 days after the index case was identified in the first six RACF outbreaks in the study area. The results indicated that rapid cohorting strategies, availability and adequate use of personal protective equipment (PPE), embedded infection control staff, and adequate outbreak preparedness plans may influence RACF containment and minimization of the spread of COVID-19 amongst residents. A summary of the literature on COVID-19 management using various quality management tools is shown in Table 1.

**Table 1:** Summary of studies on COVID-19 with operation/quality management techniques

References	Methodology / Tools used	Objective / Purpose
Bañez et al. (2020)	Six Sigma (DMAIC)	To identify factors in mitigating disease transmission
Gonella et al. (2020)	System thinking approach	To develop stock-flow diagram in healthcare services
McDermott et al. (2021)	LSS	To ensure process efficiency and patient safety
Kuiper et al. (2021)	Six Sigma	To respond to healthcare needs during the COVID-19 crisis
Hundal et al. (2021a, b)	LSS	To mitigate disruption in health care environments
Bhandar et al. (2021)	LSS, DMAIC	To ensure hospital management
Haley et al. (2021)	Systems thinking ideas	To manage social response
This Paper	Various quality management tools	To implement a system-wide integrated management plan

To date, to the best of our knowledge, no published studies have addressed the COVID-19 crisis from a system-wide viewpoint and have thus proposed integrated plans using quality management tools to achieve OE. Though many studies have focused on the medical issues of the disease, there are few studies of efficient system management within organizations (i.e., aged care facilities). Thus, this study attempts to address this research gap by examining various OE tools and techniques to contain the COVID-19 spread and to apply these in similar contexts in the future.

## Case description

COVID-19 can be spread from person to person causing flu-like symptoms, and in severe cases, may cause death. COVID-19 was recognized as a pandemic by the World Health Organization (WHO) in March 2020 and has spread to more than 200 countries and territories as of 24 October 2021 (Worldometer 2021). Although Australia was in a good position throughout the initial stage of the pandemic, as compared to other countries, it has recorded over 151,943 cases of COVID-19, including 28,5734 active cases and 1,590 deaths as of October 22, 2021, with the bulk of cases occurring within the state of Victoria. Of these, over 2797 cases have occurred within RACFs (Commonwealth of Australia, Australian Government Department of Health 2021). As of October 2021, 776 aged care residents in Australia have died from COVID-19 infection, with 684 of those deaths in aged care residents in Victoria (Department of Health [DoH], Australian Government 2021). Hence, the management of COVID-19 outbreaks in RACFs in Victoria, Australia was a significant concern.

The criticality of the COVID-19 outbreak within Victorian RACFs was identified on 22 July 2020. This outbreak went on to claim the lives of many senior Australians within one month (VACRC and the Joint Task Group 629.2 of the Australian Defence Force 2020). Soon after, the secretary of the DoH and other senior officials met to develop the best approach for responding to this situation. On 24 July 2020, a response center named the ‘Victorian Aged Care Response Centre’ (VACRC) was formed at extremely short notice to stabilize the situation, and an Executive Officer (EO) was appointed by Director General Emergency Management Australia (DGEMA) with support from the secretaries of the DoH and Home Affairs (Engineers Australia 2021).

The EO’s experience in the context of crisis and disaster management was an essential enabler of the prompt assessment of the situation and the application of crisis management principles, including the development of a concept of operations and a high-level organization structure in the VACRC. The complexity of the initial response was further exacerbated by the rapidly evolving crisis and subsequent outbreaks. Therefore, it became apparent to the EO that agility in the response time was critical and the importance of this meant that original expectations regarding governance, authorities, facilities, and systems, although important, were lower priorities.

Next, the VACRC team was formalized to commence the operation. The VACRC encompassed 21 different organizations with multiple skills, backgrounds, values, terminology, routines, and corporate cultures that needed to work together to manage the pandemic crisis in aged care facilities in Victoria. The VACRC was strengthened by a common stakeholder resolve to work to their full capacities. The VACRC quickly identified a lack of coordination between supporting agencies fueled by a lack of clearly defined roles or responsibilities. This was creating confusion across the broader aged care community.

However, COVID-19 was winning the fight. The velocity, capacity, and modes of COVID-19 spread overwhelmed the structures and the required response was beyond the systems and processes that were in place at the VACRC (then-current state). A major contributing factor to this was the lack of agility with the information system; the data were stored in silos and were not integrated across the various datasets. Real-time or near real-time risk analysis of the facilities was not possible. This was further aggravated by a lack of clarity in the process of understanding and identifying key input variables and the outcome.

The first challenge for the VACRC team was to orchestrate and develop a robust intelligence feed and a functional common operating picture. The team needed to fuse multiple streams of data and information with varying levels of coherence to assemble the picture. The team then needed to determine the next course of action to achieve the steady-state operation of the VACRC underpinned by an evidence-based decision-making process. It was necessary to shift the paradigm from Detect-to-Prevent to Predict-to-Prevent to defeat the outbreak of COVID-19 within the aged care sector of Victoria under such ambiguous and uncertain circumstances. This is the point of excellence noted in this case study. However, the volume of data, the variability within it, and the velocity at which it came from each subject matter expert (SME) varied considerably. The following critical problems were identified by the VACRC team:

- Identify key COVID-19 management inputs and clarity of process.
- Develop a system thinking approach within the aged care sector in Victoria.
- Develop a metric for holistic assessment of aged care facilities.

These needed to be addressed immediately to stabilize the situation, which subsequently set the conditions and guiding principles to achieve operational excellence.

## Methodology

The Operation COVID Assist for the Victorian aged care sector was an exemplary case study that demonstrated a true collaborative approach to predict and prevent the outbreak using the Six Sigma Methodology. The conventional, stereotype thinking with a status quo outlook was challenged and therefore a new thought-process was developed/invented. The section describes the methodology and underpinning threads required to take calculated decisions when there was no data available during ambiguous and uncertain situations (e.g., a fog of war). It details how SME knowledge was leveraged to develop multiple emergency scenarios, create narratives and subsequently transform them into:

- a valuable data to generate mathematical models,
- understand key input variables and their interactions
- predict and prevent the outbreak and
- guide the deployed force elements and minimize risk.

The initial theoretical modeling and simulations were then verified and validated with the help of real-life data (actual data from the field). Any anomalies or residues were rectified by adjusting coefficients within the mathematical model. It was also revealed that the full deployment of structured tools such as MAP (Military Appreciation Process) for the planning process was not as effective as it could be. Therefore, short sprints of multiple tools and techniques were initially trailed instead of setting a deliberate approach to tool planning.

However, this research was conducted in phases using a mixed-methods approach; this approach is becoming increasingly popular for addressing complex phenomena. According to Bryman (2007), “bringing quantitative and qualitative findings together has the potential to offer insights that could not otherwise be gleaned” (p. 9). This study commenced with a broad overview and identification of key issues through qualitative inquiries. This was followed by a focused investigation into the identified key factors using appropriate mathematical analysis tools borrowed from different disciplinary knowledge domains.

The following steps were taken to conduct the research:

### Step 1: Data collection

Within the VACRC, several agencies and authorities provided input into the decision-making process. For this study, data were collected from the following 21 stakeholder agencies who were involved in COVID-19 crisis management in Victoria:

i. DoH
ii. Australian Defence Force (ADF)
iii. Australian Medical Assistance Teams (AUSMAT)
iv. Department of Health and Human Services (DHHS)
v. Multiple third-party service providers
vi. Epidemiologists (from various hospitals)
vii. Group of surgeons (from various hospitals)
viii. Nurses (from various hospitals)
ix. Burnett Institute (an Australian medical research institute)
x. Data analytics consultants
xi. IT staff and engineers, etc.

For data collection, a section leader from each of the above sectors or agencies was selected as the SME to provide their opinion/input to the study. A checklist (see Appendix: Table 4 for details) was developed for SME compilation; this comprised over 40 entries assessing more than 20 factors. A mixed-methods approach was adopted for data collection in this real-life experimental study, with the view to developing an operational management plan to tackle the COVID-19 spread across aged care facilities in Victoria.

In this first step, a focus group discussion (FGD) following the nominal group technique (NGT) was performed to identify contributing factors and establish multiple hypotheses. The FGD method involves obtaining data from a purposely selected group of individuals, rather than from a statistically representative sample of the broader population. It is a technique where researchers gather a group of individuals to discuss a specific topic, with the aim of obtaining information based on their complex personal experiences, beliefs, perceptions, and attitudes through a moderated interaction (Nyumba et al. 2018; Smithson 2000). In contrast, the NGT, which is one of the most commonly used formal consensus development methods, involves face-to-face discussions in small groups (Harvey and Holmes 2012; McMillan et al. 2016). In this study, both FGD and NGT were applied to gain data on the opinions of SMEs to develop the models.

Step 2: Application of Analytical Hierarchical Process (AHP) In this step, the popular multi-criteria decision-making tool, Analytical Hierarchical Process (AHP) (Saaty 1994), was used to identify the weights and relative importance of the factors that were identified in the data collection process with SMEs. The details of the weights of the factors can be found in the Appendix (Table 5). Thus, the critical factors were selected to conduct the experiments. It should be mentioned that only partial usage of the AHP was deployed for decision analysis and judgment assistance for the Senior Leadership Team (SLT). Sensitivity analysis and further AHP calculations were out of scope for this case study. The following approach was taken during the partial application of the AHP:

- Set up the decision hierarchy with the help of SMEs and SLTs
- Conducted pairwise comparison of the attributes and alternatives, and finally
- Transformed the comparison into weights and checked the consistency of the decision-making and its comparison.

### Step 3: Conducting the experiment

In step 3, experiments were conducted using several quality management tools and mathematical modeling and statistical analyses were performed. The models were then validated using real-life case study data. Furthermore, the model results were adjusted with any undesirable reaction. These experiments and the corresponding results are described in detail in the next section.

**Fig. 1:**
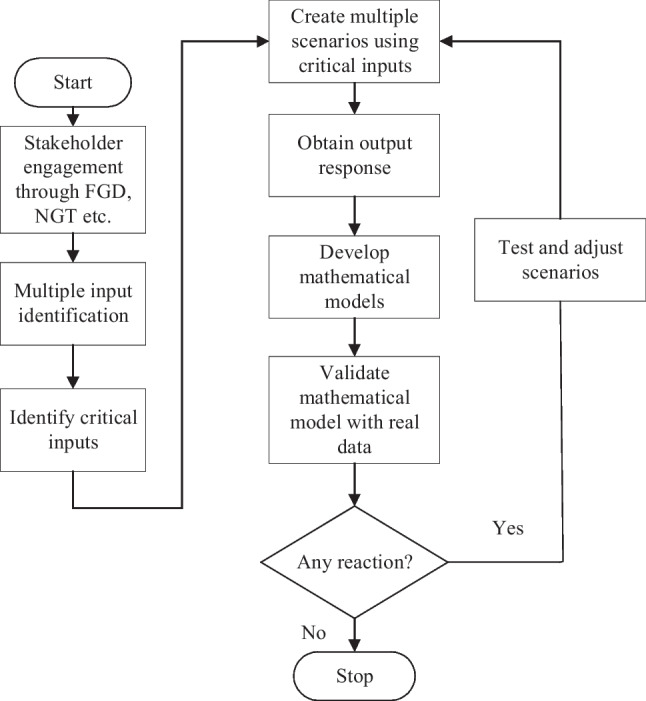
Flow chart of the Methodology

For better understanding, a flowchart of the methodology conducted in the case study is illustrated in Fig. 1.

## Experiments and results

This section presents the experiments and the results of the study using the Six Sigma (DMAIC) approach.

### Identification of key COVID -19 management Inputs and clarity of process

The first step in the experiment was to identify the major inputs responsible for the pandemic management process in aged care facilities in Victoria. This step was achieved with the following three sub-steps:

#### Phase 1: planning (DEFINE)

**Fig. 2:**
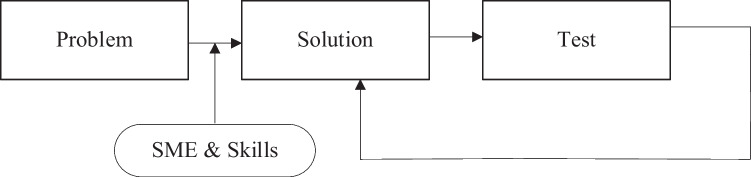
Detect and prevent approach (Allen 2020)

To accelerate knowledge discovery and identify key input and output variables involved in the achievement of the required medical effect in aged care facilities, a cross-functional stakeholder engagement activity was conducted. To challenge potential organizational bias from the stakeholder agency SMEs, a non-prescriptive approach was adopted to determine the best approaches for detecting and preventing further outbreaks. The paradigm-shifting methodology is detailed in Fig. 2.

**Fig. 3:**
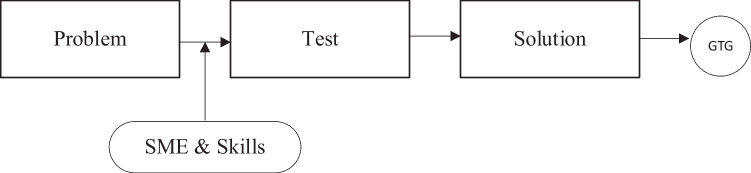
Predict and prevent approach (EA/CE forum, dated 2 Aug 21)

A checklist (see Appendix: Table 4) was developed, comprising more than 40 entries assessing more than 20 factors. However, this proved to be extremely challenging for the SMEs to provide their knowledge, with approximately 1,080,000 combinations generated by these factors. To overcome this, the approach was refined, as depicted in Fig. 3, and was applied to SME knowledge and skills to identify key variables and predict their effects.

Using the refined approach and through targeted brainstorming sessions, the following key inputs were identified.

- Leadership
- Infection, prevention and control (IPC)
- Operations management (OM; e.g., PPE, training, efficiency and effectiveness of extant processes)
- Clinical elements within the aged care facility

Next, these factors were ranked using the AHP method, as stated earlier.

**Fig. 4:**
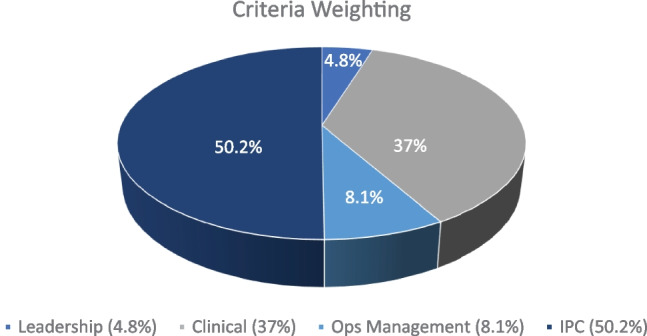
Criteria selection with the AHP method

**Fig. 5:**
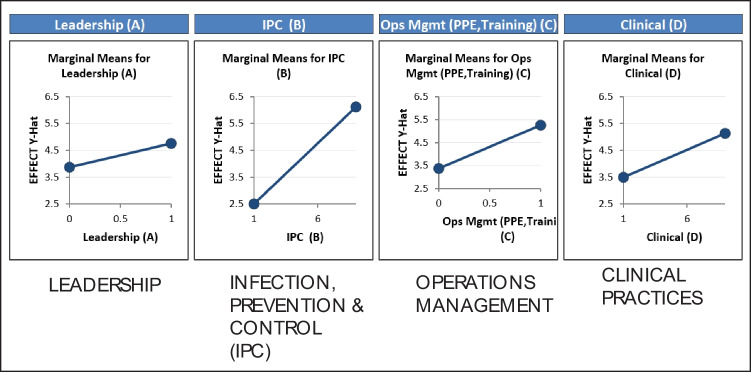
Plot of main effects obtained from the FGD

From Fig. 4, it can be seen that IPC had the highest criteria weighting (50.2%), followed by clinical elements (37%), OM (8.1%), and finally, leadership (4.8%). Thus, based on the opinions of SMEs, the most important factors were IPC and clinical elements. These findings can also be depicted as the main effects, as shown in Fig. 5. The steeper the gradient of the line, the greater the importance of the factor. It can be seen that IPC and clinical elements had the highest gradients, whereas leadership and OM were approximately equal in their gradient scores during the FGD brainstorming process.

Once the inputs were identified, measures of system performance or medical effect were developed in order to provide guidelines for the assessment of the output, including measurement, accuracy, and precision. Random replications were created using Taguchi-L8 for the DoE setup. A regression model approach was utilized to illustrate the relationships between the response, that is, the medical effect, and the input variables (i.e., leadership, IPC, OM, and clinical elements). The statistical significance of the input variables was determined using p-values of the regression model and the importance of each input variable was analyzed. In order to develop a regression model based on the significant main effects and interactions, the first step was to determine the regression coefficients.

In addition, SMEs ranked the medical effect of random combinations on a scale of 1–10, as recorded on the response table as shown in Table 2. It was considered that the unknowns could be answered more efficiently with fewer trials if the responses were repeatable, accurate, and measured on a continuous scale. It should be mentioned here that 16 scenarios were generated from Taguchi L-8, and for each, around 5 SMEs provided their opinions, which were considered as input in the study.

**Table 2:** Replication using the Taguchi L-8 technique and responses

Trials	Leadership	IPC	Ops Mgmt	Clinical	Responses 1	Responses 2
1	0	1	0	1	8	1
2	0	1	0	9	7	2
3	0	9	1	1	7	5
4	0	9	1	9	6	6
5	1	1	1	1	4	3
6	1	1	1	9	3	4
7	1	9	0	1	5	5
8	1	9	0	9	5	6
1	0	1	0	1	8	1
2	0	1	0	9	7	2
3	0	9	1	1	7	7
4	0	9	1	9	6	7
5	1	1	1	1	4	3
6	1	1	1	9	3	4
7	1	9	0	1	5	5
8	1	9	0	9	5	6

**Fig. 6:**
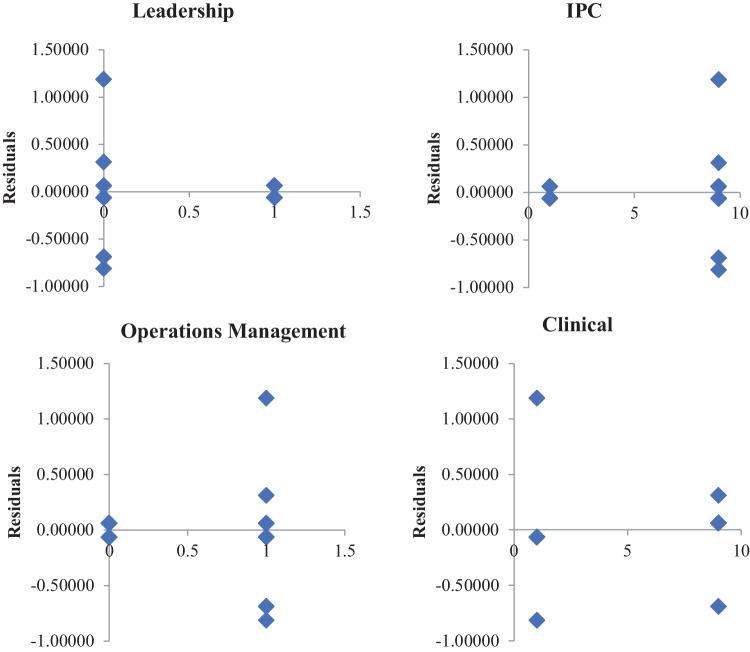
Residual charts

With the responses documented and all combinations of factors considered, the VACRC team developed several mathematical models and performed statistical analyses to develop an effective OM plan for aged care facilities. For this, a regression-based mathematical model was created using ‘Excel’, as presented in Table 3. This was created to build a predictive approach to the response function, that is, the medical effect. Whilst it was clear from the regression coefficients in Fig. 6 that leadership, IPC, and OM were more important due to their higher coefficients, it was also necessary to examine the interactions between these inputs. However, based on this analysis, it can be concluded that leadership should be considered the most important variable, with a high p-value. This contradicts the initial findings obtained from the AHP and FGD processes, as shown in Figs. 4 and 5.

**Table 3:** Mathematical model and regression analysis

Regression Statistics								
Multiple R	0.978	-						
R Square	0.956	Goodness of Fit > = 0.80	-					
Adjusted R Square	0.939	-						
Standard Error	0.494	-						
Observations	16	-						
-	-							
ANOVA	-							
-	df	SS	MS	F	P-value	-		
Regression	4	57.75	14.4375	59.09302326	0.000	-		
Residual	11	2.6875	0.244318182	-				
Total	15	60.4375	-				Confidence Level	
-						0.95	-	0.99
-	Coefficients	Standard Error	t Stat	P-value	Lower 95%	Upper 95%	Lower 99%	Upper 99%
Intercept	0.53125	0.305823567	1.737112694	0.110	-0.141863132	1.204363132	-0.41858	1.481079
Leadership	0.625	0.247142763	2.528902694	0.028	0.081042446	1.168957554	-0.14258	1.392578
IPC	0.421875	0.030892845	13.65607455	0.000	0.353880306	0.489869694	0.325928	0.517822
Ops Mgmt	1.375	0.247142763	5.563585927	0.000	0.831042446	1.918957554	0.607422	2.142578
Clinical	0.109375	0.030892845	3.540463772	0.005	0.041380306	0.177369694	0.013428	0.205322

y = 0.531 + 0.625*Leadership + 0.422*IPC + 1.375*Ops Management + 0.109*Clinical

Figure 6 presents the residual charts for all critical factors obtained from the regression analyses. The positive values for the residual (on the y-axis) mean that the prediction was too low, and the negative values mean that the prediction was too high; 0 means that the SMEs’ advice was correct. It can be seen that, aside from leadership, all other factors varied significantly between the actual and predicted values.

#### Phase 2: understanding key interactions (MEASURE)

Whilst engineers, technologists, logisticians, and professionals, in general, tend to analyze the causes of problems, the common failure is a lack of understanding of key performance input variables and, more importantly, their interactions. Therefore, in this phase, the major interactions between the input factors were studied.

**Fig. 7:**
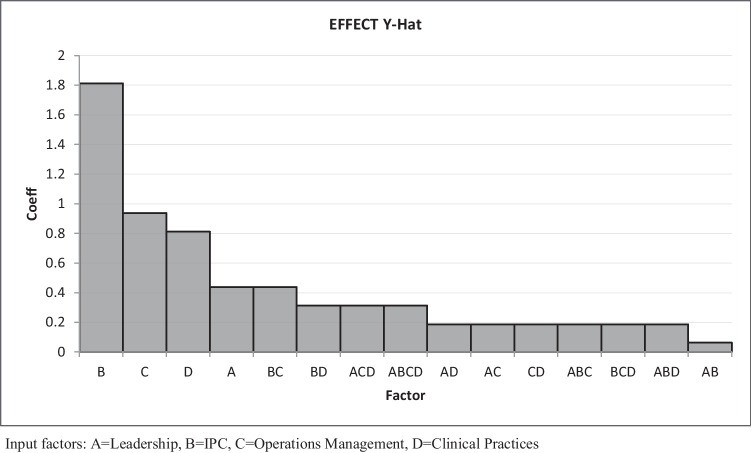
Main effects and interactions among the factors

To do this, the main effects of the above key inputs were drawn using QuantumXL software, as shown in Fig. 7. For each, the change in the output medical effect was assessed as a function of change in the input variables. IPC was seen to have the greatest impact on the medical effect, as evidenced by the steepest gradient among the four factors. However, the interaction between leadership, IPC, clinical elements, and OM was a major concern to doctors, nurses, epidemiologists, senior executives, and the whole VACRC team. As such, it required careful attention.

Thus, plots were drawn to highlight the inputs for which the interaction effects were most important to the process design and optimization study, as shown in Fig. 7. The most important input variables, their absolute values, and the interactions shown in the Pareto graph indicated that IPC was the most important factor, followed by OM, clinical elements, and leadership.

**Fig. 8:**
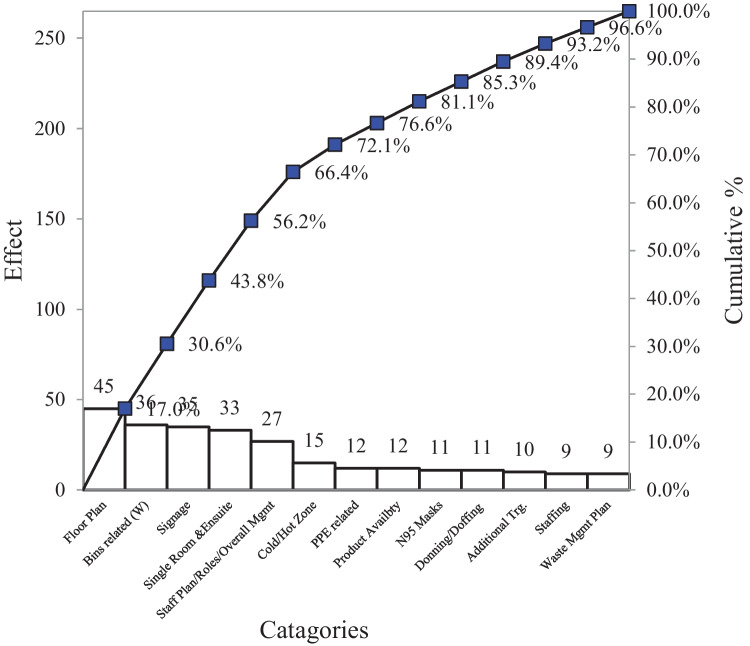
Pareto chart

**Fig. 9:**
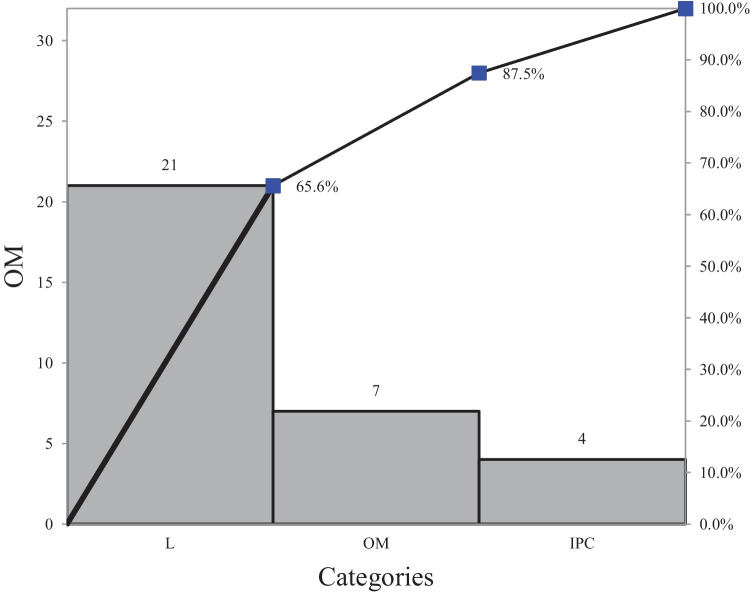
OM Pareto chart

Figure 8 shows the factors contributing to the COVID-19 outbreak within aged care centers. It can be seen in the Pareto Chart that 80% of the issues were attributed to the internal management of the centers, including the floor plan layout (17%) and bins-related (13.6%). Figure 9 presents the overall Pareto Chart for the centers. It can be seen that 87.5% of the issues within the aged care sector were directly related to leadership and OM.

Figure 9 indicates approximately 65% of the issues within the Victorian aged care centres. The raw data for this graph was through a checklist which was being audited every day for real-life field data entry. A sample checklist (Table 4) is attached in the appendix.

**Fig. 10:**
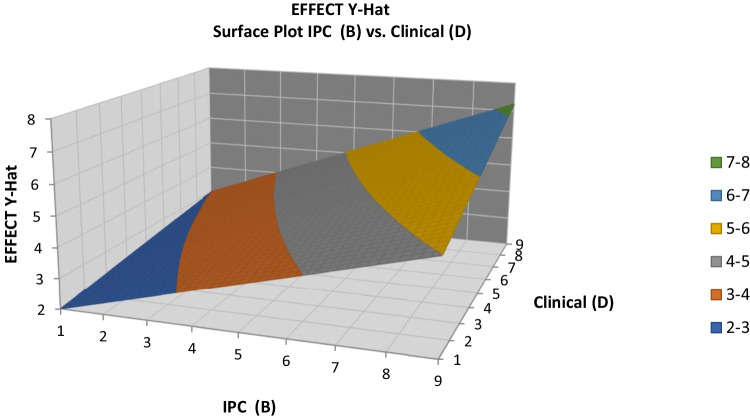
Clinical effects contour plot

**Fig. 11:**
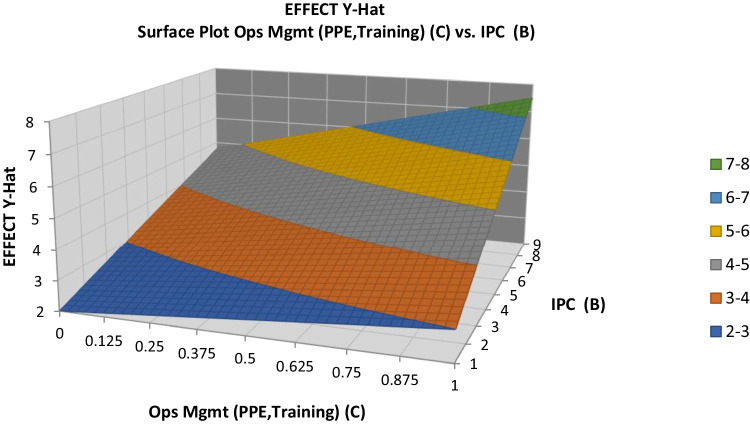
Clinical effects surface plot

Next, response contour and surface plots were drawn, as shown in Figs. 10 and 11, respectively. These graphs are useful for establishing desirable response values based on individual operating conditions. In the contour plot, the response surface is viewed as a two-dimensional plane, in which all points that have the same response are connected to produce control lines of constant responses. The surface plot displays a three-dimensional view that may provide a clearer picture of the response. The first-order regression model contains only the main effects and no interaction effects; the face of the fitted responses will be curved rather than straight. The response contour and surface plots (developed using the software Quantum XL by Sigma Zone) for the clinical effects are shown in Figs. 10 and 11, respectively. Both surface plots help to understand the nature of the relationships between IPC, OM, and clinical elements inputs and the resultant medical effects. It can be seen from the figures that the medical effect increases with increases in IPC and clinical elements, as well as with increases in IPC and OM. These factors were key contributors to enhancing the resultant medical effect.

**Fig. 12:**
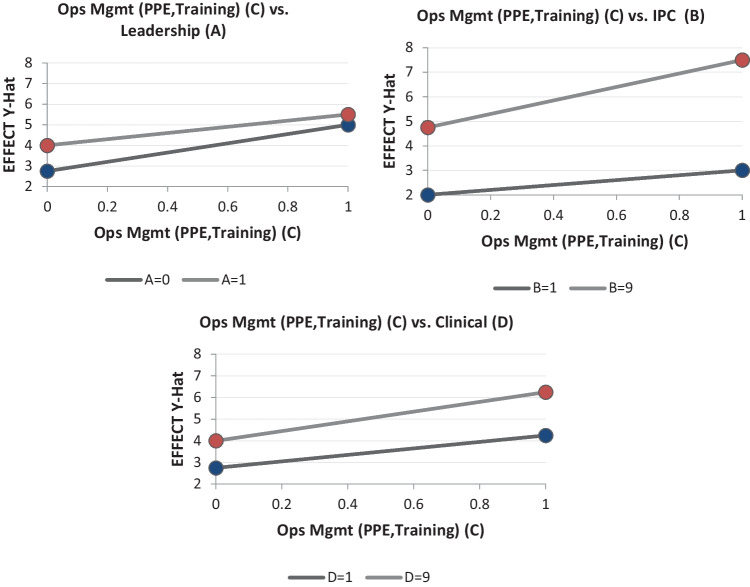
Interaction Plot and Noise Factor Plot

For more analysis, Interaction plots and Noise factors Pareto plots of the key input parameters are also derived in the study. For example, plots of parameter ‘operations management’ with others are presented in Fig. 12. It is clear from the Pareto chart that interaction between input variables leadership and operations management has the least significance or there is no interaction between leadership and operations management.

#### Phase 3: validation using control charts (ANALYSE)

**Fig. 13:**
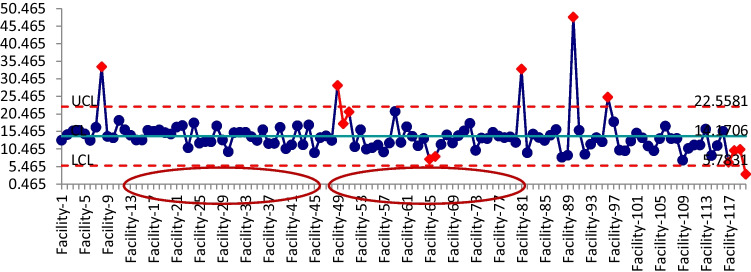
Control chart for nurse to resident ratio within aged care centers

Whilst charting data provides a simplified manner of conveying information, the use of common bar charts, pie charts, and line graphs and the trending of variables tends to depict the ‘after the fact’ or ‘for information’ data. Of greater importance is the depiction of ‘during the fact’ or ‘near real-time’ data, as this lends itself better to predictive approaches. Hence, decisions based on numerical data (or structured data) alone will rarely provide the appropriate confidence levels. For this reason, the models were validated using SME knowledge and control charts, which were then used to understand the trends, probable outbreaks, and process change within multiple clusters of aged care centers within Victoria. These data feeds were obtained through the checklist data. In this phase, a control chart was drawn using the QI Macros tool to present the average nurse to resident ratio and the control limits, as shown in Fig. 13. It can be seen that the facilities with the nurse to resident ratios below the average had a high likelihood of an outbreak, and vice versa. This predictive modeling was validated when outbreaks were predicted in at least 13 of the aged care facilities 48 h before they occurred.

In this paper, the developed mathematical model was used to source the data points for Fig. 13, which presents the nurse to resident ratio, and subsequently, these points were plotted using a control chart (also known as X-bar chart); this is another way to observe the data distribution. It should be noted that, in the chart, the X-axis represents clusters of different aged care centers and the Y-axis represents the nurse to the resident ratio (various constants). From the figure, the UCL (upper control limit) and LCL (lower control limit) indicate values three times the standard deviation from the mean value (which is the center of the line). Thus, 99.75% of the data will fall between these limits. In this situation, anything above average was most desirable, as it reflects higher patient care, whereas the below-average ratio values should be considered as unsafe conditions. Thus, the aged care centers that fell within those unsafe conditions (as highlighted with circles in the graph) were classified as high-risk for potential COVID-19 outbreak.

The control chart became a combat multiplier as it allowed potential outbreak points to be anticipated; that is, the times and places where outbreaks could possibly occur were predicted. This then allowed the force element applied to the region to be adjusted to analyze how much change was required to achieve the required clinical effect. In turn, it permitted the balancing of staff numbers within individual aged care facilities as the COVID-19 data changed rapidly. Whilst this modeling was not fully precise due to the rapidly changing pandemic and the questionable data quality, the methodology provided actionable data and analysis backed by a logical thought process that provided clarity in decision-making.

### Integrating the systems thinking approach in the aged care sector (IMPROVE)

The next step in the experiment was to develop an integrated system thinking approach for the aged care sector. When considering the integration of systems thinking approach elements in the context of Victorian aged care facilities, the cost drivers responsible for their implementation created increased pressure on individual aged care centers to become more efficient and effective. An organization may choose either a single framework or a combination of frameworks or tools to standardize its systems approach. Sound combinations predetermine a structured approach to identify problems, validate and verify problems, and analyze and solve them. In order to understand any of these frameworks, it is extremely important to understand the individual tools and techniques of each. As DMAIC is the most widely used framework and was extensively employed for LSS projects, it was used to develop the strategic excellence framework for the VACRC.

**Fig. 14:**
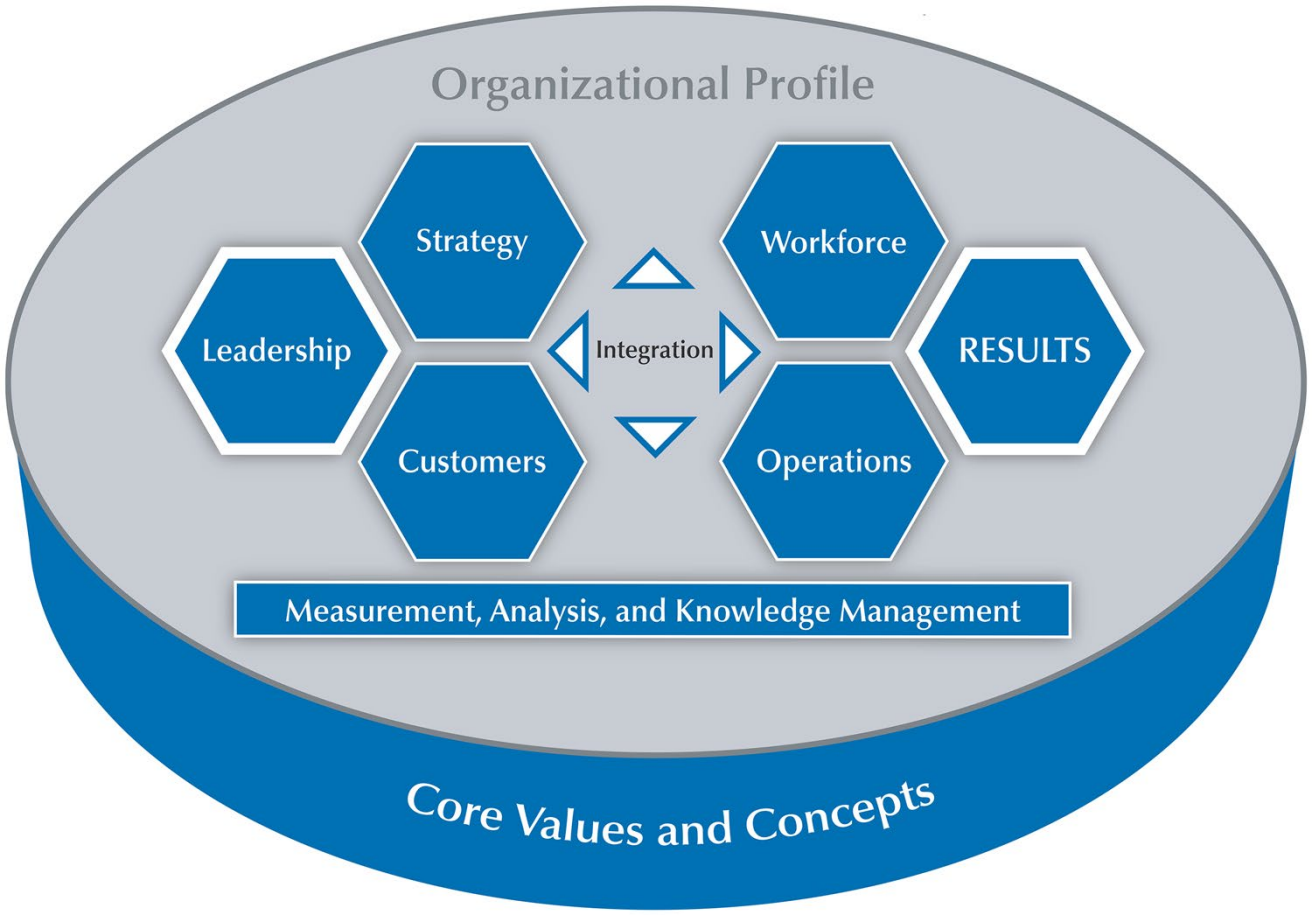
Systems approach to excellence within aged care centres

In this study, an integrated systems approach was developed to achieve OE, with an alignment to quality improvement initiatives such as LSS, as shown in Fig. 14. This may be positively complemented within the approach in the mentioned case study. In order to help leaders to build their organizations and provide customer focus excellence, the systems approach requires hard wiring of the organization context/profile (the background of organization existence), leadership, and six core elements (customer, strategy, process, employee, results), as shown in Fig. 14. The approach also consists of automatic feedback and learning loops for continuous improvement and innovation. Leadership within the organization plays a pivotal role in achieving sustainable excellence. Thus, the system commences with leadership. Next, leaders must understand customer requirements and employ strategies to meet them. End-to-end business processes must be designed for efficient and effective business outcomes. People within an organization generate results for the whole system. From these results, organizations must gain knowledge to provide feedback to the employee, help manage and improve processes, inform strategic outlooks, maintain customer relations, and help leaders to drive the business. The organizational system operates under the direction of senior leadership. As new information comes to light, feedback and learning mechanisms must distribute it promptly on a ‘need-to know’ basis. The systems approach requires leaders to understand all aspects of their business, especially through an organizational context which includes customers and products, delivery systems, employees, and governance systems, as well as competitive and strategic situations. The OE system is an underlying thread that provides a ‘conditioning and umbrella effect’ to LSS methodology.

### Holistic assessment using a metric (Control)

The last step was to introduce a holistic assessment as a metric to measure the system performance of aged care facilities. To survive and grow, an organization must meet its stated objectives. This measure can be used to identify whether an organization is meeting its objectives or not.

As business organizations, aged care facilities need to frequently measure their performance in order to evaluate their past performance, identify where the gaps are, and determine how to improve those gaps. Historically, as supply gears up to meet the rising demand for a consumer product, the productivity of an organization becomes the most important performance measure. As the gap between supply and demand decreases and competition increases, customers have better choices, and they begin demanding more features and better service. Customer demand traditionally propels companies to innovate and diversify in order to grow their businesses which, in turn, become more complex. Thus, productivity alone is an insufficient measure of business performance. Global integration and increasing competition also create challenging business dynamics. In addition to the changes brought by the COVID-19 pandemic, organizations such as aged care centers face an ever-evolving landscape of frequent collapse, mergers, acquisitions, competition from parallel and substitute products, and threats from buyers and suppliers. Excelling in this environment requires a comprehensive reporting system that can accurately read the business dynamics (Gupta 2006). Hence, a metric that can provide a holistic view of the organization and timely feedback for monitoring and improvement purposes is required.

With the current and anticipated unsettling environment (pre and post-COVID-19), aged care centers require a performance measure that is robust and that can address various aspects of the organization, including leadership, strategy, customer, operations, and processes (as detailed in Fig. 14) to provide a holistic view of organizational wellness, performance gaps, and associated risks. Therefore, an index was developed to measure the above aspects within aged care centers to assess the opportunities for improvements. The index was developed using weighted average scores for the 10 leading indicators (as shown in the figure) and their significance levels. This was performed using a consultative approach with the key stakeholders, e.g., surgeons, nurses, doctors, ADF officers, CEOs of the aged care sector, emergency services such as ambulances, etc. The higher the index, the lower the risk, and vice versa. Moreover, this index was subsequently mapped on the Six Sigma scale to understand the goodness/wellness of the organizations and to set the conditions for excellence.

**Fig. 15:**
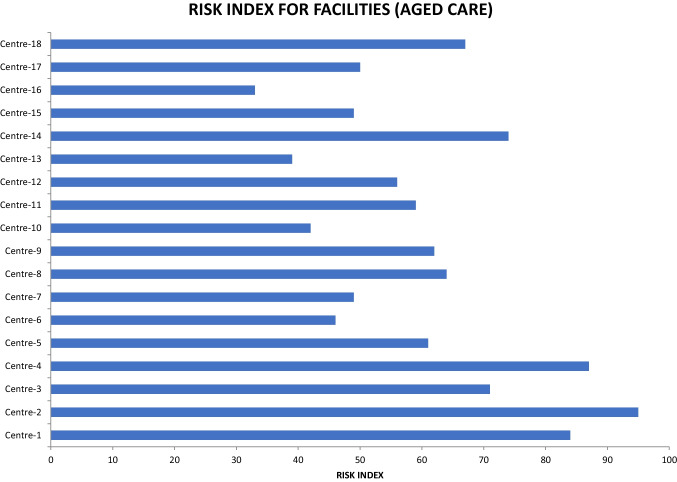
Facility wellness index and trend

An example of this index was developed using MS Excel, as shown in Fig. 15. As can be seen, there was a positive linear correlation between the wellness index and the performance of the centers. It can also be observed that a higher aged care facility wellness index means a lower risk facility risk in terms of the COVID-19 outbreak. The index threshold for aged care centers was recommended at 50 for low-performance organizations/centers.

The developed models along with the related analyses discussed above were successfully implemented in the VACRC to manage the COVID-19 crisis using LSS, systems thinking, and other relevant approaches. In this case study, several additional issues were identified that may further aggravate the situation of non-excellence. For instance: non-extant data collection regime for measurement analysis and knowledge management, a lack of relationship between operations focuses on employee and key result areas, loose connection between workforce planning and strategic planning, non-alignment of IT frameworks with business policies, etc.

## Discussion

The case study described in this paper operated under a complex, ambiguous and uncertain environment with real-world consequences for any action and, more importantly, non-action during COVID-19 crisis across Victoria. Any delay within the decision-making processes could have not only cost the lives of elderly Australians but also endangered the team’s effort. In order to stabilize the situation, a predictive approach was developed to understand the processes involved and their associated data. The predictive thinking approach, deploying LSS tools, and utilizing SME knowledge to develop robust solutions in a data-poor environment, were key contributing factors to the success of the system. In this study, qualitative data were used to develop the predictive mathematical model to manage the crisis situation. While a range of accurate quantitative data is critical, qualitative data through interviews and engaging with subjective judgment on a personal basis can be useful to complement or in the absence of numerical data. During the COVID-19 outbreak, stakeholders within the VACRC, such as medical doctors, surgeons, defense force officers, nurses, IT staff, and engineers were interviewed using FGD and NGT approaches to obtain qualitative data which were subsequently converted into more meaningful quantitative data for the purpose of further analysis. Next, the AHP was adopted to rank the criteria and several quality management tools (checklist, Six Sigma, system thinking approach, etc.) and statistical analyses (regression analysis, control chart, Pareto chart, etc.) were performed to obtain OE in the system. From this real-life experiment, it is clear that sufficient operations management measures are critical to tackling complex problems such as the COVID-19 pandemic in the aged care sector in Victoria. There is a broad consensus and clear evidence within the aged care sector context that the alignment of management functions can produce significant improvements.

### Study implications

The major contributions or implications of this study are two-fold.

- Theoretical implications

The study made significant theoretical contributions to the existing body of knowledge by;

i. offering a holistic management system, tools and techniques integrating a system thinking approach with various quality management tools for improving organizational performance in the case of challenging situations like pandemics,
ii. providing additional insight into the development of mathematical models and the use of statistical analyses to achieve OE across the system,
iii. filling the void and absence of efficient management system to deal with a complex situation with multi-stakeholder involvement and
iv. offering a single metric to assess enterprise risk and wellness.

As discussed in the literature review section and highlighted in Table 1, only a handful of research have been found applying different tools and techniques to enhance operational performances in managing wicked problems like COVID-19. However, most of these studies focused on a single tool or measure in addressing the problem scenarios. Such as Six Sigma (Bañez et al. 2020; Kuiper et al. 2021), LSS (McDermott et al. 2021; Hundal et al. 2021a, b; Bhandar et al. 2021), system thinking (Gonella et al. 2020; Haley et al. 2021) etc. have been used as operation or quality management techniques on different contexts in isolation. Hence, to the best of our knowledge, this research is the first formal study performed on a real-life case study integrating various OE tools and techniques (e.g., Six Sigma, systems thinking, checklist, etc.) and performing statistical analyses (regression analysis, control chart, Pareto chart, etc.) to develop an effective systemwide operational plan to manage critical and complex situations like COVID-19.

Inspired by the suggestions from Moxham and Kauppi (2014) and Haldorsson et al. (2015), this research used a combination of various theories and methods to solve a real-life problem by exploring some new dimensions of the operations management practice beyond the traditional OM subject areas and concepts. Thus, the study fills a gap between practice and existing theories, i.e. lack of theory-grounded research (Ketchen and Craighead 2020) in operations management, which offered a unique opportunity to understand the wicked complexity posed by COVID-19. This study is also a response to the call for theoretical concepts from other disciplines (Samson and Kalchschmidt 2019).

b) Practical implications

The study has significant implications for operations and management practitioners to tackle real-world complex, messy problems realistically. It provides an opportunity to draw lessons from the experience of VACRC, where the application of a single framework or methodology was ineffective during the COVID-19 outbreak within the aged care sector. Whilst the methodology was deployed for the Victorian aged care sector, its application in organizations could be versatile.

Taking insights from the study, practitioners can trial and deploy a combination of frameworks and methodologies in many other contexts for gaining operational efficiency. This approach departs from the 'Adhoc' and business as usual (BAU) approach, where executives or business leaders can assess and predict risks. This is one of the significant advantages of this approach. The approach and set of guiding principles for management are mostly common to organizations regardless of their type, shape, size and complexity, as all elements, including leadership, strategy, customer engagement, performance management, employee relationship, core business processes and data management are closely connected. Hence, the principles, findings, and techniques outlined in this paper can be applicable and contextualized in multiple areas.

The study will help directly the government bodies like the Department of Health of the Commonwealth of Australia to adopt and formulate a strategic framework to create a culture of organizational excellence using the Six Sigma Methodology. This is likely to help aged care sectors to take timely responses or countermeasures during the future pandemic and manage the current enterprise-wide risks. The application of this approach can be extended to profit/non-profit, education sector, hospitals, military and government organizations.

### Limitations and future research directions

The study should admit its limitations. First, it was beyond the scope of this study to trial the approach within other areas of business or industry sectors. Second, the study was conducted under a limited data availability situation and limited time frame. Hence, utilizing more data and information could make the results more robust.

While the approach detailed in this paper was deployed to the COVID-19 outbreak situation within the Victorian aged care centers, its applicability and scalability cut across other emergencies and future pandemics. Future research can be expanded by applying this approach at the strategic management level by considering all relevant aspects. Since the underlying principles, constants of management, and threads are common to any organization, the approach can be applied widely for organizational performance or excellence. In the face of constant change driven by digital transformation in organizations, more detailed studies regarding IT service architecture, technology stack, data highway and digital inequality may be undertaken using the framework detailed in this paper. The methodology of this study could also be applied in other disaster management areas as a collective decision-making frame with poor data-driven scenarios.

## Conclusion

The objective of this study was to develop an effective integrated management and measurement system to tackle the COVID-19 crisis using some contemporary quality management tools and techniques. Based on a real-life case study of several aged care facilities in Victoria, Australia, the study revealed that organizations struggled to strike a balance between a sound management system and its associated measurement systems. The study was conducted in the VACRC, where Victoria's pandemic situation was worst. The study revealed that the management system was fragmented, and the measurement system was not aligned and integrated. Thus, the approach deployed in this paper ensured the correct orchestration and synchronization of the management system and measurement system. It also 'gelled' practice and performance areas within the centers with 'hard-wiring' of the context or purpose of the organization. Thus, employing a system thinking approach in aged care centers using a single metric helped to improve organizational efficiency and effectiveness as well as compliance improvement, helping to achieve the elusive goal of OE. The key elements discussed within the paper include alignment, distributed leadership, integration, time-based, OMTM, structured thinking, customer value, predictive analytics and poor data-driven decision-making. Their strength of existence correlates strongly with superior organizational performance and risk mitigation. Executives, CEOs, and military commanders must assess, measure and improve their organizations to achieve superior performance and wellness. It is expected that once an organization has firmly established the above elements, initiatives such as quality improvement will have a solid foundational base and will likely be sustainable and successful.

In a nutshell, by developing a management system and corresponding metrics, this study explored the deeper 'pre-conditions' that explain the variance in the success of aged care centers and the resulting performance changes. This unique approach could be applied to various organizations regardless of their type, shape, and complexity. The study thus provides new insights on integrating multiple strategies that can be used in several other circumstances to achieve better outcomes.

## Appendix

**Table 4:** Checklist

Item	Input	Category	Item	Input	Category
Additional Training	10	OM		Additional Training	10
PPE Prices	4	OM	PPE	PPE-related	12
N95 Masks	11	IPC		N95 Masks	11
Staffing	9	L		Staffing	9
Funding	3	L		Signage	35
Signage	23	L	Signage	Bins-related (W)	36
High Touch Point	6	IPC		Waste Management Plan	9
Equipment Hygiene	4	IPC		Product Availability	12
Deep Clean	3	IPC		Donning/Doffing	11
Cubical Bins	9	OM	Bins-related	Staff Plan/Roles/Overall Management	27
Additional Clinical Waste Bins	14	OM	Bins-related	Cold/Hot Zone	15
Waste Management Plan	9	OM		Single Room & Ensuite	33
Signage	7	OM	Signage	Floor Plan	45
Change Between Use	5	IPC			
Product Availability	12	L			
PPE Application	4	IPC	PPE		
Instructions	6	IPC			
Waste Bins	13	OM	Bins-related		
Donning/Doffing Station	11	IPC			
PPE Compliance	4	IPC	PPE		
Availability	5	L			
Mask Face Shield	9	IPC			
Signage	5	OM	Signage		
Resident Info	4	L			
Staff Plan	17	L			
Staff to Dedicated Zone	11	L			
Cold/Hot Zones	15	IPC			
Single Room & Ensuite	33	L			
Floor Plan	45	L			
Infection Control Lead	3	IPC	L		
Dedicated Roles/Contact	7	L			
Management Plan	3	L			

**Table 5:** AHP Table

Intensity of Importance	Definition	Explanation
1	Equal Importance	Two elements contribute equally to the objective
3	Moderate Importance	Experience and judgment slightly favour one element over the other
5	Strong Importance	Experience and judgment strongly favour one element over the other
7	Very Strong Importance	One element is favoured very strongly over another, its dominance is demonstrated in practice
9	Extreme Importance	The evidence favouring one element over another is of the highest possible order of affirmation

2,4,6,8 can be used to express intermediate values
